# Myeloma‐like Castleman disease with plasmacytosis and monoclonal gammopathy

**DOI:** 10.1002/kjm2.12575

**Published:** 2022-07-26

**Authors:** Chieh‐Yu Hsieh, Chin‐Mu Hsu, Kung‐Chao Chang, Hui‐Hua Hsiao

**Affiliations:** ^1^ Division of Hematology and Oncology, Department of internal medicine Kaohsiung Medical University Hospital Kaohsiung Taiwan; ^2^ Department of pathology Kaohsiung Medical University Hospital Kaohsiung Taiwan; ^3^ Department of Pathology National Cheng Kung University Hospital, College of Medicine, National Cheng Kung University Tainan Taiwan; ^4^ Faculty of Medicine School of Medicine, Kaohsiung Medical University Kaohsiung Taiwan

To the editor:

Castleman disease, a lymphoproliferative disorder, shares specific histopathological features with variant clinical features.^1^, ^2^ Plasmacytosis is frequently found in multicentric Castleman disease, irrespective of human herpes virus (HHV8) or Epstein‐Barr virus (EBV) infection. However, marrow plasmacytosis with monoclonal gammopathy, which presents similarly to myeloma is rare.^3^

A 37‐year‐old healthy gentleman suffered from an on‐and‐off fever pattern and progressive lymph node enlargement for weeks. A physical examination showed systemic lymphadenopathy but there was no hepatosplenomegaly, neurologic abnormality, or skin lesion. A positron emission tomography‐Computed tomography scan demonstrated multiple low‐grade fluorodeoxyglucose avid lymph nodes over bilateral neck, axillary, abdominal, and inguinal areas. Biochemical tests revealed normocytic anemia (hemoglobin: 7.2 g/dl), thrombocytopenia (platelet: 62000/μl), and a reversed A/G ratio (albumin: 2.7 g/L, γ‐globulin: 3.2 g/L). An immunoglobulin (Ig) assay showed IgG: 1090 mg/L; IgA: 29.3 mg/L; IgM: 181 mg/L; kappa: 19.9 mg/L; lambda: 6.12 mg/L with a monoclonal kappa chain in immunofixation electrophoresis of urine. Viral antibody tests were only positive for cytomegalovirus‐IgG, while nonreactive to human immunodeficiency virus (HIV) or Epstein–Barr viral capsid antigen‐IgM. A bone marrow examination showed increased infiltration of plasma cells (Figure 1, 10%, left panel) without light chain restriction. A biopsy of the right axillary lymph node yielded a final diagnosis of plasma cell type Castleman disease (Figure 1, right panel) with monoclonal gammopathy of undetermined significance (MGUS), negative for HHV8 and EBV. The patient received anti‐IL6 therapy with good response and was stable for 5 years of follow‐up with loss of urine monoclonal gammopathy after therapy.^4^

**FIGURE 1:**
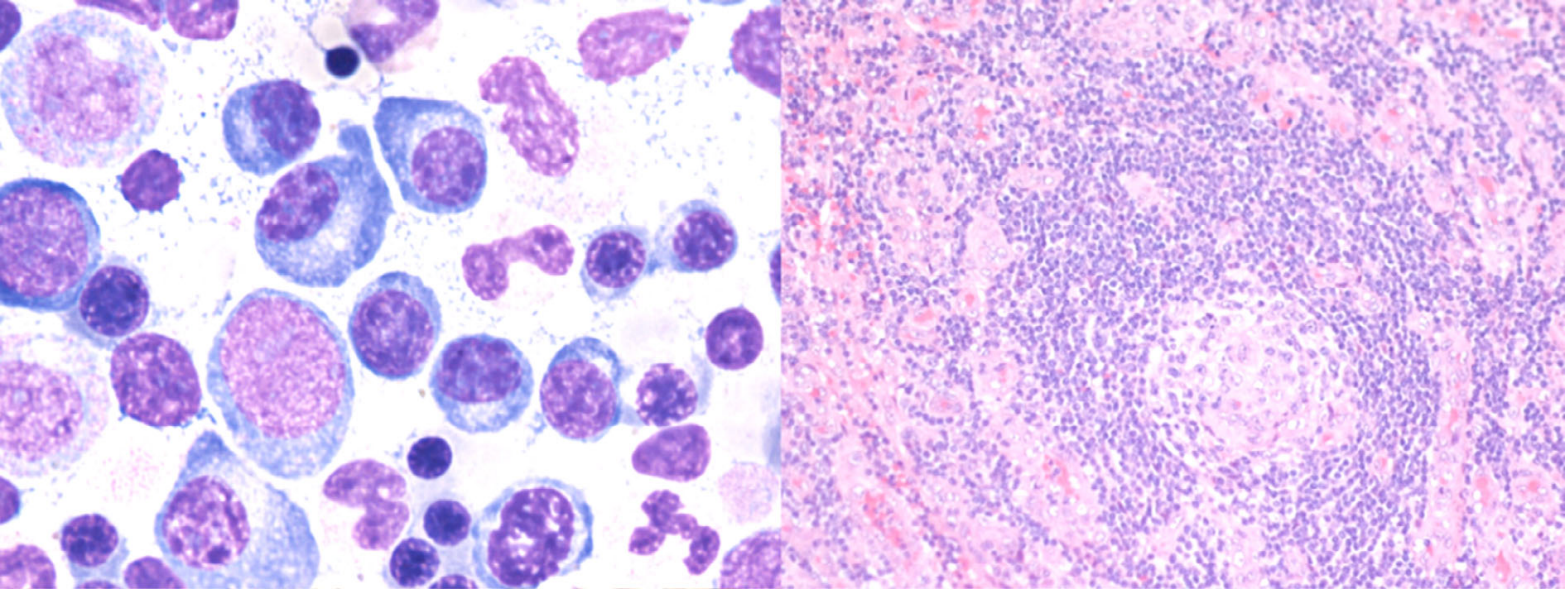
Histopathology of Castleman disease. (Left) bone marrow trephine reveals infiltration of plasma cells in the marrow. (Right) axillary lymph node biopsy presents plasma cell type‐Castleman disease with interfollicular expansion of plasma cells. The CD138 is positive on the plasma cells with polytypic of light chain staining. In addition, CD30, HHV‐8 and EBER in situ hybridization are negative, while, IL‐6 staining is positive on the plasma cells. (Left) H&E stain, 200×: (right) H&E stain, 20×

Castleman disease is a rare lymphoproliferative disorder that consists of several subtypes with heterogeneous clinical manifestations and histological features, which can hamper final diagnosis.^4^, ^5^ In addition to lymphadenopathies, some cases of multicentric Castleman disease can be found with paraneoplastic syndrome, such as POMS syndrome (polyneuropathy, orgenomegaly, endocrinopathy, M protein, skin change), similar to our case, or TAFRO syndrome (thrombocytopenia, anasarca, myelofibrosis, renal dysfunction, and organomegaly), which might lead to misdiagnosis. Fortunately, progress on disease etiologies in recent decades, shift in treatment strategies from chemotherapy to target therapy, and cytokines treatment is leading to improved patients' outcome and survival.^4^ This case highlights the importance of accurate diagnosis of Castleman disease with plasmacytosis and monoclonal gammopathy, which can be overlooked as myeloma, as well as the importance of optimal therapy for a good outcome.

## CONFLICT OF INTEREST

All authors declare no conflict of interest.
